# Supplementation with selenium and coenzyme Q_10_ in an elderly Swedish population low in selenium — positive effects on thyroid hormones, cardiovascular mortality, and quality of life

**DOI:** 10.1186/s12916-024-03411-1

**Published:** 2024-05-07

**Authors:** Urban Alehagen, Jan Alexander, Jan O. Aaseth, Anders Larsson, Trine B. Opstad

**Affiliations:** 1 Division of Cardiovascular Medicine, Department of Medical and Health Sciences, Linköping University, 581 85 Linköping, Sweden; 2 Norwegian Institute of Public Health, Oslo, Norway; 3 Department of Research, Innlandet Hospital Trust, Brumunddal, Norway; 4 Faculty of Health and Social Sciences, Inland Norway University of Applied Sciences, Lillehammer, Norway; 5 Department of Medical Sciences, Uppsala University, Uppsala, Sweden; 6 Center for Clinical Heart Research - Laboratory, Department of Cardiology, Oslo University Hospital Ullevål, Oslo, Norway; 7 Faculty of Medicine, University of Oslo, Oslo, Norway

**Keywords:** Selenium, Coenzyme Q_10_, Elderly, Thyroidal hormones, Cardiovascular mortality

## Abstract

**Background:**

Selenium-dependent deiodinases play a central role in thyroid hormone regulation and metabolism. In many European countries, insufficient selenium intake may consequently lead to adverse effects on thyroid function. In this randomised placebo-controlled double-blind study, we examined the effect of supplementation with selenium and coenzyme Q_10_ on thyroid hormonal status, cardiovascular (CV) mortality and health-related quality of life (Hr-QoL).

**Methods:**

Free T3, free T4, reverse T3, and TSH were determined in 414 individuals at baseline, and the effect of selenium yeast (200 µg/day) and coenzyme Q_10_ (200 mg/day) supplementation on hormone concentrations, CV mortality and Hr-QoL was evaluated after 48 months using Short Form 36 (SF-36). Pre-intervention plasma selenium was low, mean 67 µg/L, corresponding to an estimated intake of 35 µg/day. Changes in concentrations of thyroid hormones following the intervention were assessed using *T*-tests, repeated measures of variance, and ANCOVA analyses.

**Results:**

In the total population, the group with the lowest selenium concentration at baseline presented with significantly higher levels of TSH and lower levels of fT3 as compared to subjects with the highest selenium concentration. Supplementation with selenium and coenzyme Q_10_ for 4 years significantly increased fT3 and rT3, decreased fT4, and diminished the increase in TSH levels compared with placebo treatment (*p* = 0.03, all). In the placebo group, TSH and fT4 values above the median were associated with an increase in 10-year CV mortality, as compared with the mortality rate among those with TSH and fT4 below the median (*p* < 0.04, both), with no difference in mortality rate according to TSH and fT4 levels in the active intervention group. Similarly, TSH > median and fT3 < median were associated with a decline in mental Hr-QoL measures vs. TSH < and fT3 > median in the placebo group during 4 years of follow-up, but this was wiped out in the active group.

**Conclusions:**

Supplementation with selenium and coenzyme Q_10_ had a beneficial effect on thyroid hormones with respect to CV mortality and Hr-QoL outcomes. The initial deficient selenium status was associated with an impaired thyroid function and the changes in thyroid hormone levels can be explained by increased activity of deiodinases. We conclude that a substantial part of the elderly study population might suffer from suboptimal thyroidal function with adverse clinical implications due to selenium deficiency.

**Trial registration:**

This study was registered at ClinicalTrials.gov and has the identifier NCT01443780. Since it was not mandatory to register at the time the study began, the study has been registered retrospectively.

## Background

In the human body, the thyroid gland has the highest content of selenium in proportion to weight [1]. Correspondingly, selenium is an essential trace element for the function of the thyroidal gland, especially for the metabolism of thyroid hormones [2].

**Fig. 1:**
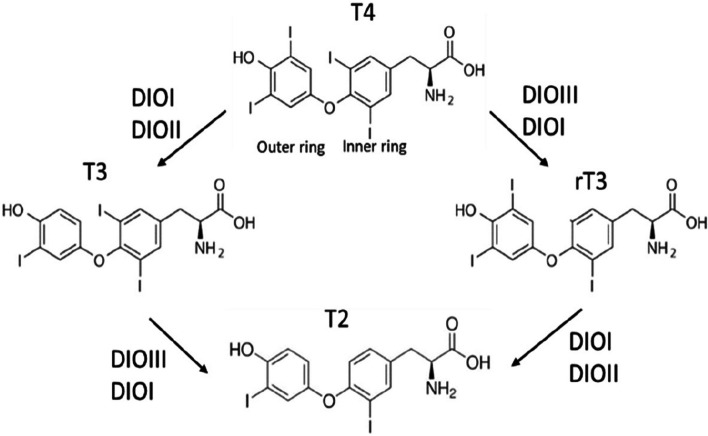
The catalytic action of the different deiodinases (DIO I, II and III) on the thyroid hormone metabolism

The effects of selenium on the thyroid gland and thyroid hormones are mediated via glutathione peroxidases (GPXs) and thioredoxin reductases (TXNRDs) that protect against oxidative cellular injury (3,6), iodothyronine deiodinases I, II and III (DIOI, DIOII and DIOIII) that activate and inactivate T4 and T3 (Fig. 1), and selenoprotein P that provides selenium to the thyroid gland and other extrahepatic tissues [3].

*Activation* of thyroid hormones occurs by conversion of T4 to the hormone T3 through the removal of an iodine atom in the outer tyrosyl ring [4] (Fig. 1). Activation is catalysed by DIOI, mainly localised in the thyroid gland, liver and kidney and by DIOII, and mainly found in extrahepatic tissue, e.g. in the brain and heart [5]. In the brain, this conversion by DIOII has a negative feedback effect via TRF on TSH release from the pituitary gland. *Inactivation* of thyroid hormones is catalysed mainly by DIOIII and occurs by the removal of an iodine atom from the inner ring, of which T4 is converted to the inactive hormone rT3 and the active T3 to inactive hormone T2 (Fig. 1).

The deiodinases play an important role in thyroid hormone central regulation and in the metabolic activity in peripheral tissues including in the heart [6, 7].

A sufficient intake of selenium is mandatory in order to avoid disturbance of the thyroid hormone balance, as an insufficient intake could affect both the central and the peripheral capacity to convert T4 to T3, causing an increased level of T4, and a reduced level of T3 [8].

In an epidemiological study from China including more than 6100 participants, a serum selenium concentration of 57.4 μg/L, which is lower than required, was associated with an increased occurrence of hypothyroidism, autoimmune thyroiditis, and an enlarged thyroid gland [9]. In a six-year follow-up study of the same population, Wu reported that an increased level of thyroid peroxidase (TPO) antibodies and a higher incidence of Hashimoto’s disease occurred in the group with low selenium intake [10]. In a meta-analysis, Toulis et al. reported significant protective effects of supplementation with selenium on the occurrence of Hashimoto´s thyroiditis [11]. In another meta-analysis including 16 trials, patients with chronic autoimmune thyroiditis treated with Levaxin and selenium supplementation had significantly reduced levels of TPO antibodies than non-supplemented patients [12]. The mechanism behind the positive effects of selenium on autoimmune thyroid disease progression is probably due to the significant effect of selenium adequacy on inflammatory mechanisms [13, 14]. However, there are also reports indicating no effect of supplementation with selenium on the thyroid function in euthyroid individuals who are almost selenium-replete [15].

In European populations, a low selenium intake is the result of low selenium content in the soil, [16, 17], whereas in the USA the selenium content in the soil is generally high. The estimated serum selenium concentrations in US citizens are generally above 120 μg/L [18, 19], as compared to the levels in several European countries, which are well below 90 μg/L [20–24], which is less than the 110 μg/L required for an optimal status [25].

Xia et al. reported an important interrelationship between selenium and coenzyme Q_10_ (ubiquinone) as TXNRD is required to obtain ubiquinol, the active form of the coenzyme [26]. The mevalonate cycle in the cell is, for optimal functioning, both dependent on an adequate supply of coenzyme Q_10_ and the synthesis of selenoproteins. An insufficiency in selenium and TXNRD activity therefore leads to suboptimal concentrations of ubiquinol in the cells. Coenzyme Q_10_ is a powerful antioxidant protecting against lipid peroxidation [27]. The endogenous production of coenzyme Q_10_ decreases after the age of 20, and the myocardial production is reduced to about half at the age of 80 [28]. Therefore, elderly people living in areas with low selenium intake may be at increased risk of cardiovascular disease (CVD) due to a possible deficiency of both these compounds.

Our research group have demonstrated higher cardiovascular (CV) mortality in an elderly community population with low plasma selenium concentration, and a reduced CV mortality following supplementation with selenium and coenzyme Q_10_ [29]. Additionally, health-related quality of life (Hr-QoL) was improved [30]. In the literature, less attention has been paid to the possible role of low selenium in thyroid hormone regulation with respect to CV morbidity and Hr-QoL.

The aim of the present sub-study was to examine associations between serum selenium concentrations and levels of the thyroid hormones and to determine if intervention with selenium and coenzyme Q_10_ beneficially influences the thyroid hormone concentrations and further CV mortality and Hr-QoL.

## Methods

### Subjects

This sub-study was conducted on a population recruited from a municipality in the south-east of Sweden where all inhabitants aged 70–88 were invited to participate in an epidemiological project in 1996. Out of the 1130 individuals in the appropriate age stratum, 875 agreed to participate in the epidemiological project. In 2003, a new invitation was sent out to the participants and of the 675 still living in the municipality, 443 agreed to participate in a dietary supplementation project that required taking selenium and coenzyme Q_10_ combined for 4 years. The inclusion started in January 2003 and finished in February 2010.

Before starting the intervention, the selenium concentration in the population was found to be 67 μg/L (standard deviation (SD 16.8)), which approximated to a daily intake of about 35 μg/day, which is far below the level considered necessary for optimal physiological function (≥ 110 μg/L) and adequate production of selenoproteins [31].

The participants received 200 mg/day of coenzyme Q_10_ capsules (Bio-Quinon 100 mg B.I.D, Pharma Nord, Vejle, Denmark) and 200 µg/day of organic selenium yeast tablets (SelenoPrecise 100 µg B.I.D, Pharma Nord, Vejle, Denmark), or placebo (500 mg vegetable oil supplied with vitamin E and bakers’ yeast, respectively) over 48 months. The supplementation was taken in addition to any regular medication. All study medications (active drug and placebo) not consumed were returned and counted as a measure of compliance.

In this sub-analysis, 210 individuals were randomised to active intervention, while 204 individuals were randomised to placebo. All participants with a known, or suspected thyroidal disease, or on medication for a thyroidal disease were excluded from this sub-analysis.

At inclusion, one of three experienced cardiologists examined all the participants, a new medical history was recorded, a new clinical examination was performed, and assessment of cardiac functional class according to the New York Heart Association (NYHA) classification was performed. Blood pressure was measured, and electrocardiograms and Doppler-echocardiograms were obtained. The echocardiographic examination was performed with the participant in the left lateral position, and the cardiac ejection fraction (EF) was categorised into four classes, with the following inter-class readings: 30%, 40% and 50% [32, 33]. A normal cardiac function was defined as EF ≥ 50%, while severely impaired systolic function was defined as EF < 30%. Only the systolic function was evaluated.

The exclusion criteria for the main project were: recent myocardial infarction; planned CV operative procedure within 4 weeks; hesitation concerning whether the candidate could decide for him/herself to participate in the study or not, or doubt about whether he/she understood the consequences of participation; a serious disease that substantially reduced survival or when it was not expected that the participant could cooperate for the full 4-year period; other factors making participation unreasonable, or drug/alcohol abuse [34]. CV mortality was defined as mortality due to myocardial infarctions, cerebrovascular lesions, fatal cardiac arrhythmias, heart failure and aortic aneurysms.

CV mortality was registered for the study participants for a follow-up period of 10 years. Mortality information was obtained from the National Board of Health and Welfare in Sweden, which registers all deaths of Swedish citizens based on death certificates or autopsy reports.

At baseline and 48 months, data on Hr-QoL was collected using the generic Short Form 36 (SF-36) [30]. SF-36 is a generic HR-QoL questionnaire which consists of 36 items that are transformed into eight domains of HR-QoL: physical functioning (PF); role limitations due to physical health problems (RP); bodily pain (BP); general health (GH); vitality (VT); social functioning (SF); role limitations due to emotional health problems (RE); and mental health (MH). Subsequently, these domains are aggregated into two composite scores: physical component score (PCS) and mental component score (MCS) (22). The PCS comprises the domains PF, RP, BP and GH, whereas the MCS includes VT, SF, RE and MH (23). The scores are transformed into values of 0–100, with a higher score indicating a better HR-QoL.

All patients provided written informed consent.

#### Ethical approval

The study was approved by the Regional Ethical Committee (Forskningsetikkommmitten, Hälsouniversitetet, SE-581 85 Linköping, Sweden; No. D03-176), and conforms to the ethical guidelines of the 1975 Declaration of Helsinki. (The Medical Product Agency declined to review the study protocol since the study was not considered a trial of a medication for a certain disease but rather one of food supplement commodities that are commercially available). This study was registered at ClinicalTrials.gov and has the identifier NCT01443780. Since it was not mandatory to register at the time the study began, the study has been registered retrospectively.

### Biochemical analyses

Blood samples were collected at the start of the study and after 48 months, and drawn with the participants resting in a supine position. Pre-chilled EDTA vials for the collection of plasma were used. The vials were centrifuged at 3000* g*, + 4 °C, and the EDTA plasma samples were frozen at − 70 °C. No sample was thawed more than once.

### Determination of the thyroid hormones

Thyroglobulin (kit: DuoSet ELISA DY8306, R&D Systems, Minneapolis, MN, USA), free T3 (kit: T3F31-K01), free T4 (kit: T4F31-K01), reverse T3 (kit: RT331-K01), and TSH (kit: THH31-K01), were analysed using commercial ELISA kits (Eagle Biosciences, Amherst, NH, USA).

### Statistical methods

Descriptive data are presented as percentages or mean ± standard deviation (SD). A Student’s unpaired two-sided *T*-test was used for continuous variables and the chi-square test was used for analysis of one discrete variable. Repeated measures of variance were used to obtain better information on the individual changes in the concentration of the biomarker analysed, compared to group mean values.

Analysis of covariance (ANCOVA) evaluation was performed on both log_10_ transformed and non-transformed data, with no significant difference in the results.

In the ANCOVA evaluation, the actual biomarker concentration after 48 months was used as a dependent variable. In the model, adjustments were made for several variables that are either well-known to influence CV mortality, or to potentially covariate with the biomarker analysed. Thus, the variables that were adjusted for varied for the different thyroid hormones, as seen in the respective tables. *P*-values < 0.05 were considered significant, based on a two-sided evaluation. All data were analysed using standard software (Statistica v. 13.2, Dell Inc, Tulsa, OK).

## Results

From the baseline characteristics, it is obvious that the active treatment group and the placebo group are well balanced, and no significant differences were noted in any of the covariables (Table 1).

**Table 1:** Baseline characteristics of the study population divided into active treatment and placebo

	**Active treatment**	***P*-value**	**Placebo**
***N***	210		204
Age years mean (SD)	77.0 (3.6)	0.30	77.3 (3.4)
Sex; males/females ***n*** (%)	112/98		104/100
**History**			
Smokers ***n*** (%)	19 (9.0)	0.80	17 (8.3)
Diabetes ***n*** (%)	46 (21.9)	0.78	47 (23.0)
Hypertension ***n*** (%)	150 (71.4)	0.55	151 (74.0)
IHD ***n*** (%)	45 (21.4)	0.61	48 (23.5)
NYHA class I ***n*** (%)	111 (52.9)	0.38	99 (48.5)
NYHA class II ***n*** (%)	59 (28.1)	0.85	59 (28.9)
NYHA class III ***n*** (%)	39 (18.6)	0.52	43 (21.1)
NYHA class IV ***n*** (%)	0		0
Unclassified NYHA ***n*** (%)	1		3
**Medications**			
ACEI ***n*** (%)	33 (15.7)	0.79	34 (16.7)
ARB ***n*** (%)	9 (4.3)		11 (5.4)
Betablockers ***n*** (%)	76 (36.2)	0.41	66 (32.7)
Digitalis ***n*** (%)	10 (4.8)		9 (4.4)
Diuretics ***n*** (%)	66 (31.4)	0.47	71 (34.8)
Statins ***n*** (%)	43 (20.5)	0.69	45 (22.1)
**Examinations**			
EF < 40% ***n*** (%)	16 (7.6)		16 (7.8)
s-selenium pre-intervention µg/L, mean (SD)	66.7 (16.0)	0.80	66.3 (18.1)
s-selenium post-intervention µg/L, mean (SD)	210.2 (60.7)	< 0.00001	71.9 (25.6)
ECG sr incl, ***n*** (%)	155/210 (73.8)	0.40	143/204 (70.1)
ECG sr end, ***n*** (%)	150/173 (86.7)	0.95	140/161 (87.0)
ECG AF incl, ***n*** (%)	14/210 (6.7)	0.57	15/204 (7.4)
ECG AF end, ***n*** (%)	18/173 (10.4)	0.82	18/161 (11.2)
ECG status pos infarction incl, ***n*** (%)	10/210 (4.8)	0.36	14/204 (7.4)
ECG status post infarction end, ***n*** (%)	17/173 (9.8)	0.56	19/161 (11.8)

*ACEI* Angiotensin converting enzyme inhibitor, *AF* Atrial fibrillation, *ARB* Angiotensin receptor blockers, *ECG* Electrocardiogram, *EF* Ejection fraction, *IHD* Ischemic heart disease, *NYHA* New York Heart Association functional class, *sr* Sinus rhythm

In the total study population, 301 participants (72.7%) had hypertension, 93 (22.5%) had diabetes, 49 (11.8%) had atrial fibrillation, 93 (22.5%) had ischaemic heart disease, and 32 (7.7%) presented with impaired cardiac function. The obtained results are in line with what could be expected in a group of Scandinavians of corresponding age. The measured concentrations of selenium were low in both groups and corresponded to an approximate intake of about 35 µg/day, thus obviously lower than the recommended guidelines. In the total population included in the present study, no significant difference in concentration of fT3 and TSH was observed in subjects with atrial fibrillation or in those with sinus rhythm (2.79 pg/mL vs. 2.76 pg/mL; *p* = 0.86 and 1.56 µlU/mL vs. AF: 1.31 µlU/mL; *p* = 0.10, respectively).

### Levels of the thyroid hormones in relation to selenium status at baseline

Selenium concentrations were categorised into quartiles (Qs) and the distribution of the thyroid hormones through them was evaluated and further compared between Q1 (lowest selenium values) and Q4 (highest selenium values). A significantly higher TSH concentration was observed in those in the first quartile compared to those in the fourth quartile (Q1: 1.72 µlU/mL, vs. Q4: 1.43 µlU/mL; *p* = 0.023), whereas no difference was observed between thyroglobulin (Q1: 22.84 ng/mL vs Q4: 19.35 ng/mL; *p* = 0.53). Significantly higher levels of fT3 were found in the group with the highest selenium concentration vs. the lowest (Q1: 2.50 pg/mL vs. Q4: 2.75 pg/mL; *p* = 0.004). In the group with lower fT3 (below median), we noted significantly higher CRP levels than in those above median (< median: 8.31 mg/L vs > median: 3.53 mg/L; *p* = 0.04). No significant differences in concentrations of rT3 and fT4 were seen in those with a higher level of selenium, as compared with those with lower concentration (Q1: 0.09 pg/mL vs. Q4: 0.10 pg/mL; *p* = 0.30 and 8.07 pg/mL vs. 8.80 pg/mL; *p* = 0.081, respectively).

### TSH and fT4 in relation to cardiovascular mortality

CV mortality after 10 years was evaluated according to TSH below versus above the median value at baseline (1.38 µIU/ml in the total population). In the placebo group, significantly higher CV mortality was observed in the group of participants with a TSH value above the median in comparison with those below the median (< median: *n* = 34 out of 103 vs. > median: *n* = 47 out of 100; chi^2^: 4.16; *p* = 0.042). Such a difference in CV mortality was not seen in the group of participants receiving the active treatment (< median: *n* = 22 out of 105 vs. > median: *n* = 22 out of 105).

Regarding fT4, a similar difference was shown; the participants in the placebo group with a fT4 concentration above median presented with significantly higher CV mortality, compared to those with a fT4 below median (> median: *n* = 47 out of 101 vs < median: *n* = 33 out of 102, *p* = 0.039), with no difference in the active treatment group (> median: 53 out of 105 vs < median: 50 out of 105, *p* = 0.63).

## Effects on thyroid hormones of selenium and coenzyme Q_10_ supplementation

### TSH in relation to supplementation with selenium and coenzyme Q_10_

At the start of the intervention, there was no significant difference in mean TSH concentration between the active and the placebo groups (active: 1.56 µlU/mL vs. placebo: 1.49 µlU/mL; *p* = 0.48), and this remained the case after 48 months of intervention (active: 1.74 µlU/mL vs. placebo: 2.00 µlU/mL; *p* = 0.14). When analysing the change in TSH between inclusion and after 48 months, we found a significant increase in the placebo group (incl: 1.49 µlU/mL vs. 48 months: 2.00; *p* = 0.001), whereas there was no significant change in the active group (incl: 1.56 µlU/mL vs. 48 months: 1.74 µlU/mL; *p* = 0.11). The percentage of individuals exceeding 4.2 µIU/ml.

(which is the cut-off used to indicate subclinical hypothyroidism) was 2.2% in the total study population at inclusion, which in the placebo group increased from 2.5% to 9.6%, whereas a minimal change was observed in the active group (from 1.9% to 3.4%) after 4 years.

**Fig. 2:**
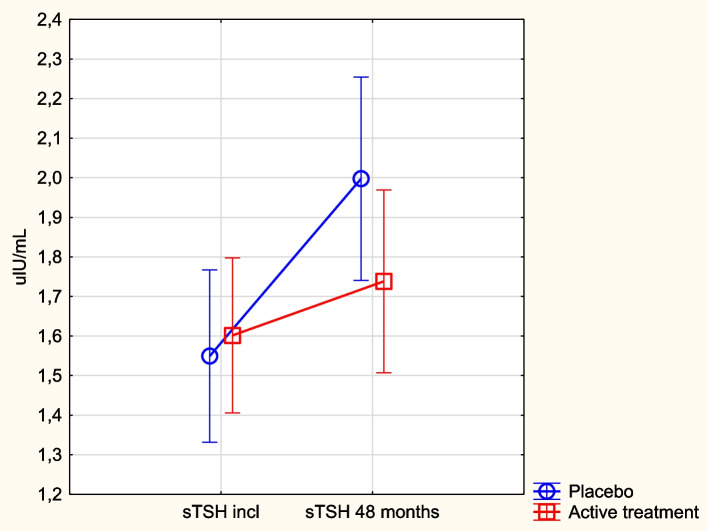
Concentration of TSH at inclusion and after 48 months in the selenium and coenzyme Q_10_ treatment group compared to the placebo group in the study population. Note: Evaluation performed by use of repeated measures of variance methodology. Note: Current effect: F(1, 206) = 4.54; *p* = 0.034. Note: Blue circles: placebo; red squares: active treatment group. Bars indicate ± 95% confidence interval (CI)

Performing repeated measures of variance analysis, a significantly smaller increase in TSH was observed in the active group (*p* = 0.03) (Fig. 2). The ANCOVA analysis, where adjustments were made for several important clinical factors, showed that besides the intervention with selenium and coenzyme Q_10_, both the pre-intervention concentration of TSH and selenium showed a significant influence on the 48-month level of TSH (Table 2).

**Table 2:** Analysis of covariance using TSH after 48 months as dependent variable

Effects	Sum of squares	*F*	*P*
Intercept	0.0092	0.009	0.92
TSH incl	121.35	119.43	< 0.0001
BMI	0.05	0.05	0.83
NT-proBNP	0.37	0.37	0.54
Age	0.002	0.002	0.96
Smoker	0.12	0.12	0.72
Active treatment	5.15	5.15	0.03
Hypertension	0.06	0.06	0.82
Diabetes	0.004	0.004	0.95
IHD	0.29	0.29	0.59
Hb < 120 g/L	1.82	1.82	0.18
EF < 40%	0.37	0.37	0.54
s-selenium pre-intervention µg/L	6.27	6.27	0.014
Error	194.07		

*BMI* Body mass index, *EF* Ejection fraction, *IHD* Ischemic heart disease, *NT-proBNP* N-terminal fragment of B-type natriuretic peptide

### fT3 in relation to supplementation with selenium and coenzyme Q_10_

At inclusion, there was no significant difference between the active treatment and the placebo groups (active: 2.76 pg/mL vs. placebo: 2.77 pg/mL; *p* = 0.97). After 48 months, there was a significantly higher level of fT3 in the active treatment group compared with the placebo group (active: 3.03 pg/mL vs. placebo: 2.73 pg/mL; *p* = 0.03). In the active treatment group, we observed a significant increase in fT3 after 48 months (incl: 2.76 pg/mL vs. 48 months: 3.03 pg/mL; *p* = 0.02), whereas there was no significant change in the concentration in the placebo group (2.77 pg/mL vs. 2.73 pg/mL; *p* = 0.78).

**Fig. 3:**
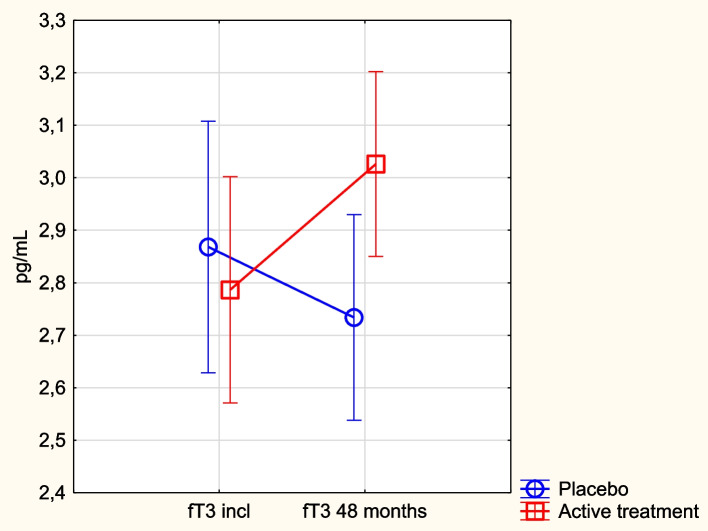
Concentration of fT3 at inclusion and after 48 months in the selenium and coenzyme Q_10_ treatment group compared to the placebo group in the study population. Note: Evaluation performed by use of repeated measures of variance methodology. Note: Current effect: F(1, 206) = 4.76; *p* = 0.030. Note: Blue circles: placebo; red squares: active treatment group. Bars indicate ± 95% CI

Applying repeated measures of variance, we found a significantly higher concentration in the active treatment group during the intervention time (*p* = 0.03) (Fig. 3). Validating the result with ANCOVA, we found that only fT3 and the active treatment significantly influenced the outcome (Table 3).

**Table 3:** Analysis of covariance using fT3 after 48 months as dependent variable

Effects	Sum of squares	*F*	*P*
Intercept	0.39	0.46	0.50
fT3 incl	20.74	24.54	< 0.0001
BMI	1.00	1.18	0.28
NT-proBNP	0.58	0.69	0.41
Age	0.004	0.004	0.95
Smoker	0.05	0.06	0.81
Active treatment	5.04	6.03	0.01
Hypertension	0.06	0.07	0.79
Diabetes	0.06	0.07	0.79
IHD	0.05	0.06	0.80
Hb < 120 g/L	0.54	0.64	0.43
EF < 40%	0.01	0.01	0.91
s-selenium pre-intervention µg/L	0.17	0.20	0.66
Error	161.39		

*BMI* Body mass index, *EF* Ejection fraction, *IHD* Ischemic heart disease, *NT-proBNP* N-terminal fragment of B-type natriuretic peptide

### rT3 in relation to supplementation with selenium and coenzyme Q_10_

At inclusion, we found no significant difference in rT3 concentration between the active treatment and the placebo groups (active: 0.091 pg/mL vs. placebo: 0.091 pg/mL; *p* = 0.90). After 48 months, we noted a significantly higher concentration of rT3 in the active treatment group than in the placebo group (active: 0.12 pg/mL vs. placebo; 0.09 pg/mL; *p* = 0.03). A significant change in concentration was seen in the active treatment group (inclusion: 0.091 pg/mL vs. 48 months: 0.122 pg/mL; *p* = 0.006), with no significant change in the placebo group (incl: 0.091 pg/mL vs. 48 months: 0.090 pg/mL; *p* = 0.98).

**Fig. 4:**
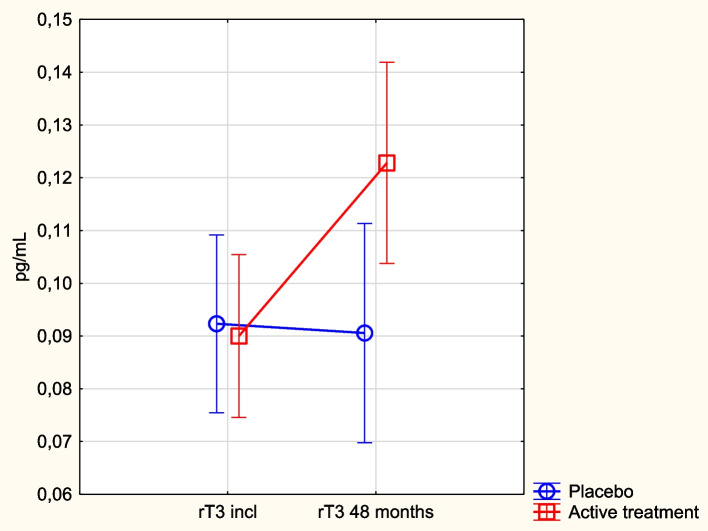
Concentration of rT3 at inclusion and after 48 months in the selenium and coenzyme Q_10_ treatment group compared to the placebo group in the study population. Note: Evaluation performed by use of repeated measures of variance methodology. Note: Current effect: F(1, 193) = 4.95; *p* = 0.027. Note: Blue circles: placebo; red squares: active treatment group. Bars indicate ± 95% CI

In the evaluation through repeated measures of variance, a significant difference between the active and the placebo groups could be noted (*p* = 0.027) (Fig. 4). In the subsequent ANCOVA evaluation, only active treatment had a significant impact, besides the inclusion level of rT3 (Table 4).

**Table 4:** Analysis of covariance using rT3 after 48 months as dependent variable

Effects	Sum of Squares	*F*	*P*
Intercept	0.002	0.26	0.61
rT3 incl	0.16	17.00	< 0.0001
BMI	0.03	2,80	0.10
NT-proBNP	0.015	1.69	0.20
Age	0.002	0.26	0.61
Smoker	0.01	1.22	0.27
Active treatment	0.06	6.32	0.01
Hypertension	0.004	0.41	0.52
Diabetes	0.003	0.28	0.60
IHD	0.01	1.45	0.23
Hb < 120 g/L	0.0007	0.08	0.78
EF < 40%	0.004	0.46	0.50
s-selenium pre-intervention µg/L	0.005	0.53	0.47
Error	1.63		

*BMI* Body mass index, *EF* Ejection fraction, *IHD* Ischemic heart disease, *NT-proBNP* N-terminal fragment of B-type natriuretic peptide

### fT4 in relation to supplementation with selenium and coenzyme Q_10_

**Fig. 5:**
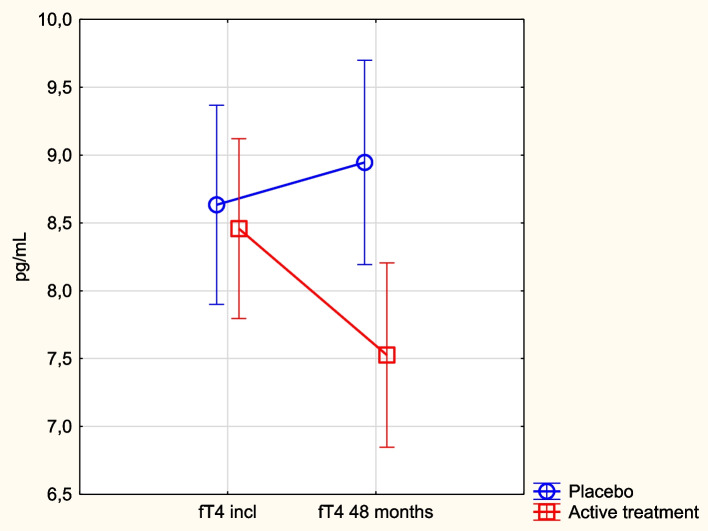
Concentration of fT4 at inclusion and after 48 months in the selenium and coenzyme Q_10_ treatment group compared to the placebo group in the study population. Note: Evaluation performed by use of repeated measures of variance methodology. Note: Current effect: F(1, 205) = 5.07; *p* = 0.025. Note: Vertical bars denote 0.95 CI. Note: Blue circles: placebo; red squares: active treatment group. Bars indicate ± 95% CI

At inclusion, there was no significant difference in fT4 concentration between the active treatment and the placebo groups (active: 8.81 pg/mL vs. placebo: 8.77 pg/mL; *p* = 0.91). After 48 months, a highly significant difference in fT4 level could be noted (active: 7.53 pg/mL vs. placebo: 8.88 pg/mL; *p* = 0.009). A significant change in concentration of fT4 was seen in the active group (incl: 8.81 pg/mL vs. 48 months: 7.53 pg/mL; *p* = 0.0003) whereas no significant change was noted in the placebo group (incl: 8.77 pg/mL vs. 48 months: 8.88 pg/mL; *p* = 0.83). Following the change in the individual values through repeated measures of variance, we found a significant difference between the active treatment and the placebo groups (*p* = 0.025) (Fig. 5). In the subsequent ANCOVA evaluation both the covariates active treatment and Hb < 120 g/L significantly influenced the result, besides the inclusion level of fT4 (Table 5).

**Table 5:** Analysis of covariance using fT4 after 48 months as dependent variable

Effects	Sum of squares	*F*	*P*
Intercept	9.04	3.27	0.07
fT4 incl	81.88	29.59	< 0.0001
BMI	0.18	0.07	0.80
NT-proBNP	1.47	0.53	0.47
Age	4.16	1.50	0.22
Smoker	6.80	2.46	0.12
Active treatment	18.13	6.55	0.01
Hypertension	8.72	3.15	0.08
Diabetes	2.72	0.98	0.32
IHD	2.74	0.99	0.32
Hb < 120 g/L	14.10	5.10	0.03
EF < 40%	6.36	2.30	0.13
Error	525.74		

*BMI* Body mass index, *EF* Ejection fraction, *IHD* Ischemic heart disease, *NT-proBNP* N-terminal fragment of B-type natriuretic peptide

Considering the deiodinase activity, we examined the impact of the intervention on the fT3/fT4 ratio and found a significant difference between those on active treatment versus those on placebo (active treatment: 0.48 vs. placebo: 0.38; *p* = 0.028), which concurs with the obtained results above.

### Thyroid hormones and association with health-related quality of life

**Fig. 6:**
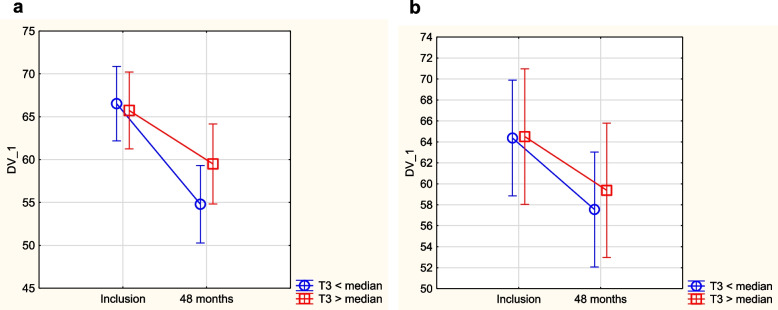
Evaluation of dimension “vitality” from the quality-of-life questionnaire SF-36 in the study population divided into those having a fT3 level below median concentration vs. those with a fT3 level above median concentration. **a** Placebo group. Note: Current effect: F(1,182) = 4.39, *p* = 0.038. Note: Vertical bars denote 0.95 CI. **b** Active treatment group. Note: Current effect: F(1,93) = 0.19, *p* = 0.66. Note: Vertical bars denote 0.95 CI

**Fig. 7:**
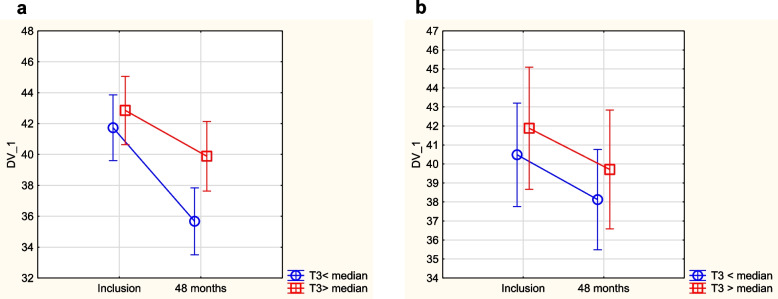
Evaluation of dimension “physical composite score” from the quality-of-life questionnaire SF-36 in the study population divided into those having a fT3 level below median concentration vs. those with a fT3 level above median concentration. **a** Placebo group. Note: Current effect: F(1,167) = 5.01, *p* = 0.03. Note: Vertical bars denote 0.95 CI. **b** Active treatment group. Note: Current effect: F(1,89) = 0.01, *p* = 0.92. Note: Vertical bars denote 0.95 CI

**Fig. 8:**
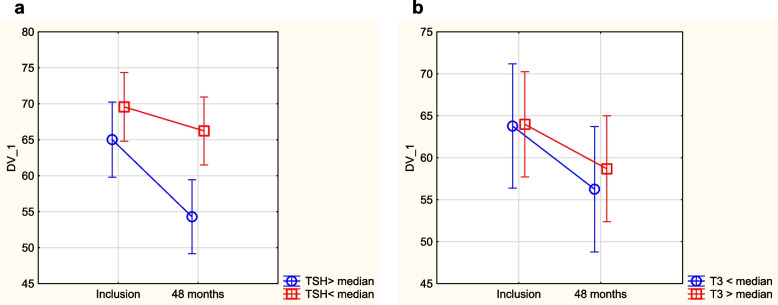
Evaluation of dimension “bodily pain” from the quality-of-life questionnaire SF-36 in the study population divided into those having a TSH level below median concentration vs. those with a TSH level above median concentration. **a** Placebo group. Note: Current effect: F(1,186) = 4.57, *p* = 0.03. Note: Vertical bars denote 0.95 CI. **b** Active treatment group. Note: Current effect: F(1,94) = 0.21, *p* = 0.64. Note: Vertical bars denote 0.95 CI

**Fig. 9:**
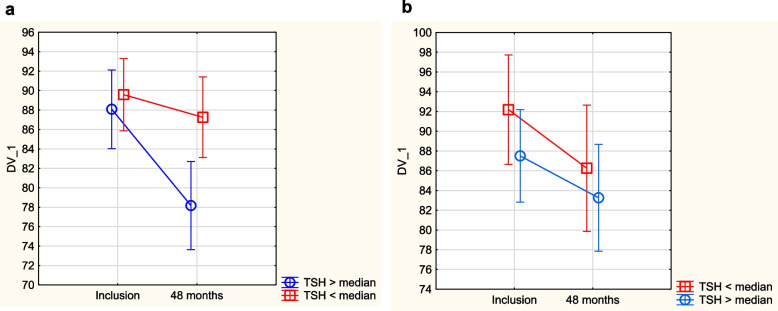
Evaluation of dimension “social function” from the quality-of-life questionnaire SF-36 in the study population divided into those having a TSH level below median concentration vs. those with a TSH level above median concentration. **a** Placebo group. Note: Current effect: F(1,186) = 5.18, *p* = 0.02. Note: Vertical bars denote 0.95 CI. **b** Active treatment group. Note: Current effect: F(1,94) = 0.14, *p* = 0.71. Note: Vertical bars denote 0.95 CI

As impaired thyroid function might be related to reduced quality of life, we evaluated possible associations between Hr-QoL symptoms and relatively low fT3 and high TSH. From the routine clinical medical record, we did not find any symptoms indicating a possible relation to impaired thyroid function. However, when assessing Hr-QoL measures with the SF-36 questionnaire, we found that those with a TSH value > median or a fT3 value < median in the placebo group had a worse quality of life in the mental dimensions “vitality”, “bodily pain”, “social function”, and in the composite dimension “physical composite score” over a period of 4 years (Figs. 6, 7, 8 and 9). A corresponding difference in the scores between those above vs. below the median of fT3 or TSH was not seen in the group given supplementation. No other Hr-QoL dimensions showed any significant differences in those with values above vs below the median of fT3 or TSH, respectively.

## Discussion

In the present intervention study on elderly Swedes low in selenium, we found that thyroid hormone plasma concentrations, i.e. TSH and fT3, but not fT4, rT3 or thyroglobulin were related to selenium plasma concentration at baseline. Low selenium concentrations were associated with high TSH and low fT3 levels. In the placebo group, elevated TSH levels at baseline were related to an increase in 10-year CV mortality. Low fT3 concentration was associated with a higher degree of inflammation as measured by CRP. The intervention lasting for 48 months resulted in an increase in fT3 and rT3 and a decrease in fT4, with no significant changes in the placebo group.

The changes in thyroid hormones accord well with the assumption that low selenium status is associated with a decreased deiodinase activity, both in the thyroid gland and in the liver, and other peripheral tissues. Following intervention, an increase in DIOI and DIOII activity would be compatible with the observed increase in fT3, decrease in fT4 and increased fT3/fT4 ratio in plasma, and a lower pituitary TSH secretion due to the negative feedback of fT3. The increased rT3 concentration accords with increased DIOIII activity.

There have been several reports in the literature that hypothyroid patients often suffer from a low-grade inflammation and increased oxidative stress, which is interesting from a mechanistic point of view. Tellechea [35] showed, in a meta-analysis including 93 studies, that in patients with hypothyroidism, it was possible to risk-stratify the patients according to CRP concentration. Gu et al. reported in their study, which included more than 3100 patients comprising more than 7700 patient-years, an increased risk for carotid atherosclerosis related to low-grade systemic inflammation [36]. Lasa and Contreras-Jurado argued in their review, that one explanation for this association could be the modulating function of thyroid hormones on inflammatory pathways, through their binding to specific nuclear thyroid receptors, all pointing to the close association between the thyroid hormones and inflammation [37]. This concurs with our finding of higher CRP concentration in the group with the lowest fT3 levels. Furthermore, several recent publications [38, 39], also from our group [14, 40, 41], point to a close relationship between inflammation, oxidative stress and selenium intake. From our main study, of which the present is a sub-study, we reported reduced CV mortality, increased cardiac systolic function, signs of less inflammatory activity and reduced oxidative stress in the intervention group compared with the placebo group [40, 42, 43].

In the present study, individuals with low selenium status presented with a significantly higher concentration of TSH and higher CV mortality, compared to those with higher selenium concentrations. It is tempting to suggest that the effect on CV mortality might, in part, have been mediated through an impact on thyroid hormones as well as a beneficial effect of selenium and coenzyme Q_10_ on inflammation and oxidative stress.

An important finding of this study is that less severe selenium deficiency may also impair the activity of selenium-dependent deiodinases, which is crucial in the metabolism and homeostasis of thyroid hormones. Hence, it might be suggested that a significant proportion of “healthy” community-living elderly participants may suffer from impaired thyroid function beyond “subclinical hypothyroidism”. Supplementation with selenium appears not only to restore deiodinase activity and thyroid hormone balance but also has clinical implications in terms of its association with reduced CV morbidity and inflammation. It has been reported that patients with a subclinical hypothyroidism have a higher risk of developing CVD as compared to subjects with normal thyroid function [44].

We have also assessed different dimensions of Hr-QoL as obtained by the quality of life questionnaire SF-36, one of the most widely used generic Hr-QoL questionnaires in the world. From the evaluations, we were able to demonstrate symptoms, mainly concerning the mental dimension, in those with impaired thyroid function. The presence of symptoms that were not identified by the routine medical record, raises the question of whether the participants had a “subclinical” condition. The scores obtained are in the range of a healthy Swedish population at the corresponding age [45]. However, after 4 years, the group with a TSH value > median or a fT3 value < median worsened their scores in the four dimensions mentioned and had clearly lower dimension scores compared to a healthy Swedish reference population of corresponding age. It is also of interest to note that in the group receiving active treatment, the differences in dimension scores between high and low TSH and fT3 appeared to be wiped out, presumably mediated by the resident effect supplementation has on the thyroid hormones.

In the literature, a debate is ongoing concerning whether subclinical hypothyroidism should be treated. In patients with substantial CV risk, there seems to be a consensus that it is advisable to start treatment [46–48]. Based on our results, it is tempting to recommend assessing the selenium status, and if low, start supplementation and observe whether the thyroid hormone status is improved *before* a hormonal treatment. In our study, we observed that a low selenium intake was associated with elevated TSH levels, which, although not exceedingly high, were in turn associated with significantly higher CV mortality within 10 years of observation, which concurs with the literature.

The obtained results are interesting and add to the knowledge on the different impacts and mechanisms of selenium status on human physiology. However, the results should be regarded as hypothesis-generating, and should stimulate further research.

## Limitations

The investigated study population consists of a relatively narrow age stratum; thus, it is not possible or appropriate to extrapolate the results to other age groups.

The sample size of the study population is relatively small, which increases the uncertainty of the obtained results. We have therefore used a two-step validation and based on that, we consider the results most likely to be accurate. Nevertheless, based on the small sample size the results are regarded as hypothesis-generating.

The evaluated population consisted of Caucasians who were low in selenium. Therefore, the obtained results could not be applied to other ethnicities without uncertainty. Neither would they be applicable to selenium-replete populations.

Another limitation is that we did not have any ultrasound examinations of the thyroidal glands of the participants, and therefore do not have information on changes in size or structure of the gland following the intervention. While we have no specific knowledge about the iodine intake in the study population, the iodine intake in Sweden is generally sufficient, provided iodised salt is used [49]. Also, as our study is an epidemiological project with a CV profile, a specific anamnesis directed towards possible symptoms of hypothyroidism was not taken; however, the medical history was a general history and included general symptoms.

## Conclusions

In this sub-analysis, “healthy” elderly community-living persons low in selenium were evaluated regarding their thyroidal function in relation to selenium intake and CV morbidity. Elevated TSH and low fT3 levels were observed in those with the lowest selenium intake, and selenium/coenzyme Q_10_ supplementation resulted in significantly increased fT3 and decreased fT4 levels. Lower TSH levels were associated with reduced CV mortality and improved measures of Hr-QoL. The observed changes in thyroid hormones could be explained by an increase in selenium-dependent deiodinases. We conclude that a substantial part of the study population might suffer from suboptimal thyroid function due to an example of an insufficient selenium intake. However, more research is needed to validate the obtained results.

## Data Availability

Under Swedish Law, the authors cannot share the data used in this study and cannot conduct any further research other than what is specified in the ethical permissions application. For inquiries about the data, researchers should first contact the owner of the database, the University of Linköping. Please contact the corresponding author with requests for and assistance with data. If the university approves the request, researchers can submit an application to the Regional Ethical Review Board for the specific research question that the researcher wants to examine.

## Abbreviations

ANCOVA: Analysis of covariance
BP: Bodily pain
CRP: C-reactive protein
CV: Cardiovascular
CVD: Cardiovascular disease
DIOs: Iodothyronine deiodinases
EF: Ejection fraction
GH: General health
GPXs: Glutathione peroxidases
Hr-QoL: Health-related quality of life
MCS: Mental component score
MH: Mental health
NYHA: New York Heart Association functional classification
PCS: Physical component score
PF: Physical functioning
Qs: Quartiles
RE: Role limitations due to emotional health problems
RP: Role limitations due to physical health problems
SD: Standard deviation
SF: Social functioning
T3: 3,5,3′-Triiodthyronine
T4: Thyroxin
TPO: Thyroid peroxidase
TRF: Thyreotropin-releasing factor
TXNRD: Thioredoxin reductases
VT: Vitality

## References

[1] Aaseth J, Frey H, Glattre E, Norheim G, Ringstad J, Thomassen Y (1990). Selenium concentrations in the human thyroid gland. Biol Trace Elem Res.

[2] Ventura M, Melo M, Carrilho F (2017). Selenium and Thyroid Disease: From Pathophysiology to Treatment. Int J Endocrinol.

[3] Kohrle J (2021). Selenium in Endocrinology-Selenoprotein-Related Diseases, Population Studies, and Epidemiological Evidence. Endocrinology.

[4] Dentice M, Salvatore D (2011). Deiodinases: the balance of thyroid hormone: local impact of thyroid hormone inactivation. J Endocrinol.

[5] Croteau W, Davey JC, Galton VA, St Germain DL (1996). Cloning of the mammalian type II iodothyronine deiodinase. A selenoprotein differentially expressed and regulated in human and rat brain and other tissues. J Clin Invest..

[6] Al-Mubarak AA, van der Meer P, Bomer N (2021). Selenium, Selenoproteins, and Heart Failure: Current Knowledge and Future Perspective. Curr Heart Fail Rep.

[7] Barreiro Arcos ML (2022). Role of thyroid hormones-induced oxidative stress on cardiovascular physiology. Biochim Biophys Acta Gen Subj.

[8] Schomburg L, Kohrle J (2008). On the importance of selenium and iodine metabolism for thyroid hormone biosynthesis and human health. Mol Nutr Food Res.

[9] Wu Q, Rayman MP, Lv H, Schomburg L, Cui B, Gao C, Chen P, Zhuang G, Zhang Z, Peng X (2015). Low Population Selenium Status Is Associated With Increased Prevalence of Thyroid Disease. J Clin Endocrinol Metab.

[10] Wu Q, Wang Y, Chen P, Wei J, Lv H, Wang S, Wu Y, Zhao X, Peng X, Rijntjes E (2022). Increased Incidence of Hashimoto Thyroiditis in Selenium Deficiency: A Prospective 6-Year Cohort Study. J Clin Endocrinol Metab.

[11] Toulis KA, Anastasilakis AD, Tzellos TG, Goulis DG, Kouvelas D (2010). Selenium supplementation in the treatment of Hashimoto's thyroiditis: a systematic review and a meta-analysis. Thyroid.

[12] Wichman J, Winther KH, Bonnema SJ, Hegedus L (2016). Selenium Supplementation Significantly Reduces Thyroid Autoantibody Levels in Patients with Chronic Autoimmune Thyroiditis: A Systematic Review and Meta-Analysis. Thyroid.

[13] Winther KH, Rayman MP, Bonnema SJ, Hegedus L (2020). Selenium in thyroid disorders - essential knowledge for clinicians. Nat Rev Endocrinol.

[14] Alehagen U, Alexander J, Aaseth J, Larsson A (2019). Decrease in inflammatory biomarker concentration by intervention with selenium and coenzyme Q_10_: a subanalysis of osteopontin, osteoprotergerin, TNFr1, TNFr2 and TWEAK. J Inflamm (Lond).

[15] Rayman MP, Thompson AJ, Bekaert B, Catterick J, Galassini R, Hall E, Warren-Perry M, Beckett GJ (2008). Randomized controlled trial of the effect of selenium supplementation on thyroid function in the elderly in the United Kingdom. Am J Clin Nutr.

[16] Rayman MP (2012). Selenium and human health. Lancet.

[17] U.S. Department of Agriculture ARS: Nutrient Intakes from Food: Mean amounts conusmed per individual, one day, 2005–2006. wwwarsusdagov /ba/bhnrc/fsrg Accessed March 2010 2008.

[18] Kafai MR, Ganji V (2003). Sex, age, geographical location, smoking, and alcohol consumption influence serum selenium concentrations in the USA: third National Health and Nutrition Examination Survey, 1988–1994. J Trace Elem Med Biol.

[19] Bleys J, Navas-Acien A, Laclaustra M, Pastor-Barriuso R, Menke A, Ordovas J, Stranges S, Guallar E (2009). Serum selenium and peripheral arterial disease: results from the national health and nutrition examination survey, 2003–2004. Am J Epidemiol.

[20] Van Cauwenbergh R, Robberecht H, Van Vlaslaer V, Deelstra H (2004). Comparison of the serum selenium content of healthy adults living in the Antwerp region (Belgium) with recent literature data. J Trace Elem Med Biol.

[21] Burri J, Haldimann M, Dudler V (2008). Selenium status of the Swiss population: assessment and change over a decade. J Trace Elem Med Biol.

[22] Letsiou S, Nomikos T, Panagiotakos D, Pergantis SA, Fragopoulou E, Antonopoulou S, Pitsavos C, Stefanadis C (2009). Serum total selenium status in Greek adults and its relation to age. The ATTICA study cohort. Biol Trace Elem Res.

[23] Spina A, Guallar E, Rayman MP, Tigbe W, Kandala NB, Stranges S (2013). Anthropometric indices and selenium status in British adults: the U.K. National Diet and Nutrition Survey. Free Radic Biol Med.

[24] Galan-Chilet I, Tellez-Plaza M, Guallar E, De Marco G, Lopez-Izquierdo R, Gonzalez-Manzano I, Carmen Tormos M, Martin-Nunez GM, Rojo-Martinez G, Saez GT (2014). Plasma selenium levels and oxidative stress biomarkers: A gene-environment interaction population-based study. Free Radic Biol Med.

[25] Alexander J, A-K O: Selenium - a scoping review for Nordic Nutrition Rcommendations 2023. Food Nutr Res 2023, 67. 10.29219/fnr.v67.10320 PMC10770655 38187789

[26] Xia L, Nordman T, Olsson JM, Damdimopoulos A, Bjorkhem-Bergman L, Nalvarte I, Eriksson LC, Arner ES, Spyrou G, Bjornstedt M (2003). The mammalian cytosolic selenoenzyme thioredoxin reductase reduces ubiquinone. A novel mechanism for defense against oxidative stress. J Biol Chem.

[27] Bullon P, Roman-Malo L, Marin-Aguilar F, Alvarez-Suarez JM, Giampieri F, Battino M, Cordero MD (2015). Lipophilic antioxidants prevent lipopolysaccharide-induced mitochondrial dysfunction through mitochondrial biogenesis improvement. Pharmacol Res.

[28] Kalen A, Appelkvist EL, Dallner G (1989). Age-related changes in the lipid compositions of rat and human tissues. Lipids.

[29] Alehagen U, Johansson P, Bjornstedt M, Rosen A, Post C, Aaseth J (2016). Relatively high mortality risk in elderly Swedish subjects with low selenium status. Eur J Clin Nutr.

[30] Johansson P, Dahlstrom O, Dahlstrom U, Alehagen U (2015). Improved Health-Related Quality of Life, and More Days out of Hospital with Supplementation with Selenium and Coenzyme Q_10_ Combined. Results from a Double Blind, Placebo-Controlled Prospective Study. J Nutr Health Aging.

[31] Blomhoff R, Andersen R, Arnesen KE, J.J. C, Eneroth H, Erkkola M, Gudanaviciene L, Halldorsson I, Höyer-Lund A, Warensjö-Lemming E (2023). Nordic Nutrition Recommendations 2023; Integrating Environmental Aspects. Copenhagen: Nordisk Minierråd.

[32] Jensen-Urstad K, Bouvier F, Hojer J, Ruiz H, Hulting J, Samad B, Thorstrand C, Jensen-Urstad M (1998). Comparison of different echocardiographic methods with radionuclide imaging for measuring left ventricular ejection fraction during acute myocardial infarction treated by thrombolytic therapy. Am J Cardiol.

[33] van Royen N, Jaffe CC, Krumholz HM, Johnson KM, Lynch PJ, Natale D, Atkinson P, Deman P, Wackers FJ (1996). Comparison and reproducibility of visual echocardiographic and quantitative radionuclide left ventricular ejection fractions. Am J Cardiol.

[34] Alehagen U, Johansson P, Bjornstedt M, Rosen A, Dahlstrom U (2013). Cardiovascular mortality and N-terminal-proBNP reduced after combined selenium and coenzyme Q_10_ supplementation: a 5-year prospective randomized double-blind placebo-controlled trial among elderly Swedish citizens. Int J Cardiol.

[35] Tellechea ML (2021). Meta-analytic evidence for increased low-grade systemic inflammation and oxidative stress in hypothyroid patients. Can levothyroxine replacement therapy mitigate the burden?. Endocrine.

[36] Gu Y, Meng G, Zhang Q, Liu L, Wu H, Zhang S, Wang Y, Zhang T, Wang X, Sun S (2022). Association of longitudinal trends in thyroid function with incident carotid atherosclerosis in middle-aged and older euthyroid subjects: the Tianjin Chronic Low-Grade Systemic Inflammation and Health (TCLSIH) cohort study. Age Ageing.

[37] Lasa M, Contreras-Jurado C (2022). Thyroid hormones act as modulators of inflammation through their nuclear receptors. Front Endocrinol (Lausanne).

[38] Farrokhian A, Bahmani F, Taghizadeh M, Mirhashemi SM, Aarabi MH, Raygan F, Aghadavod E, Asemi Z (2016). Selenium Supplementation Affects Insulin Resistance and Serum hs-CRP in Patients with Type 2 Diabetes and Coronary Heart Disease. Horm Metab Res.

[39] Asemi Z, Jamilian M, Mesdaghinia E, Esmaillzadeh A (2015). Effects of selenium supplementation on glucose homeostasis, inflammation, and oxidative stress in gestational diabetes: Randomized, double-blind, placebo-controlled trial. Nutrition.

[40] Alehagen U, Lindahl TL, Aaseth J, Svensson E, Johansson P (2015). Levels of sP-selectin and hs-CRP Decrease with Dietary Intervention with Selenium and Coenzyme Q_10_ Combined: A Secondary Analysis of a Randomized Clinical Trial. PLoS ONE.

[41] Dunning BJ, Bourgonje AR, Bulthuis MLC, Alexander J, Aaseth JO, Larsson A, van Goor H, Alehagen U (2023). Selenium and coenzyme Q(10) improve the systemic redox status while reducing cardiovascular mortality in elderly population-based individuals. Free Radic Biol Med.

[42] Alehagen U, Aaseth J, Johansson P (2015). Less increase of copeptin and MR-proADM due to intervention with selenium and coenzyme Q_10_ combined: Results from a 4-year prospective randomized double-blind placebo-controlled trial among elderly Swedish citizens. BioFactors.

[43] Alehagen U, Aaseth J, Alexander J, Svensson E, Johansson P, Larsson A (2017). Less fibrosis in elderly subjects supplemented with selenium and coenzyme Q_10_-A mechanism behind reduced cardiovascular mortality?. Biofactors.

[44] Bielecka-Dabrowa A, Godoy B, Suzuki T, Banach M, von Haehling S (2019). Subclinical hypothyroidism and the development of heart failure: an overview of risk and effects on cardiac function. Clin Res Cardiol.

[45] Sullivan M, Karlsson J (1998). The Swedish SF-36 Health Survey III. Evaluation of criterion-based validity: results from normative population. J Clin Epidemiol.

[46] Redford C, Vaidya B (2017). Subclinical hypothyroidism: Should we treat?. Post Reprod Health.

[47] Calissendorff J, Falhammar H (2020). To Treat or Not to Treat Subclinical Hypothyroidism, What Is the Evidence?. Medicina (Kaunas).

[48] Calsolaro V, Niccolai F, Pasqualetti G, Tognini S, Magno S, Riccioni T, Bottari M, Caraccio N, Monzani F (2019). Hypothyroidism in the Elderly: Who Should Be Treated and How?. J Endocr Soc.

[49] Nystrom HF, Brantsaeter AL, Erlund I, Gunnarsdottir I, Hulthen L, Laurberg P, Mattisson I, Rasmussen LB, Virtanen S, Meltzer HM (2016). Iodine status in the Nordic countries - past and present. Food Nutr Res.

